# Sink Node Placement and Partial Connectivity in Wireless Sensor Networks †

**DOI:** 10.3390/s23229058

**Published:** 2023-11-09

**Authors:** Yun Wang

**Affiliations:** Computer Science and Information Systems, Bradley University, Peoria, IL 61625, USA; ywang2@bradley.edu

**Keywords:** full connectivity, sink placement, partial connectivity, sensor connection rate, sink placement, wireless sensor networks

## Abstract

This research delves into the aspects of communication and connectivity problems within random Wireless Sensor Networks (WSNs). It takes into account the distinctive role of the sink node, its placement, and application-specific requirements for effective communication while conserving valuable network resources. Through mathematical modeling, theoretical analysis, and simulation evaluations, we derive, compare, and contrast the probabilities of partial and full connectivity within a random WSN, factoring in network parameters and the maximum allowable hop distance/count $h_{max}$. $h_{max}$ captures the diverse range of delay-sensitive requirements encountered in practical scenarios. Our research underscores the significant impact of the sink node and its placement on network connectivity and the sensor connection rate. The results exemplify a noteworthy decline in the sensor connection rate, dropping from 98.8% to 72.5%, upon relocating the sink node from the network center to the periphery. Moreover, as compared with full connectivity, partial connectivity and the sensor connection rate are more suitable metrics for assessing the communication capability of random WSNs. The results illustrate that 1.367 times *more* energy is required to connect less than 4% of the remote sensors, based on the examined network settings. Additionally, to increase the sensor connection rate slightly from 96% to 100%, an additional 538% more energy is required in multipath fading based on the widely adopted energy consumption model. This research and its outcomes contribute to establishing appropriate performance metrics and determining critical network parameters for the practical design and implementation of real-world wireless sensor networks.

## 1. Introduction

Network connectivity as a fundamental issue in Wireless Sensor Networks (WSNs) has been attracting researchers’ attention for decades, primarily focusing on strategies to achieve or maintain full connectivity for communication in diverse applications [1,2,3,4,5,6]. For full connectivity, there exists at least one communication path connecting any pair of nodes in the network. The isolation of one single node will render the resulting network disconnected [7]. In addition, a network is said to be *k*-connected if the removal of any (k−1) sensors does not render the resulting network disconnected [8]. However, sensor isolation is not an exception but rather a state of normality in practice [9,10]. The underlying reasons include, but are not limited to, workload variations, non-uniform data communication, unbalanced energy consumption, different sensor capabilities, and so on. Thus, investigating the properties of a partially connected WSN and examining the inter-relationships between network parameters and the network performance while economizing network resources is an imperative task.

Furthermore, the current literature on communication and connectivity analysis predominantly focuses on ad hoc network models where all nodes are treated as equivalent. The crucial and unique role of the sink node, responsible for data collection and fusion, has not received the attention it deserves [7] in data-centric, power-restricted, delay-sensitive, and fault-tolerant WSNs [11]. To be specific, a sensor’s connection status depends upon its ability to establish a pathway to reach the sink node for data fusion. Unlike ad hoc networks, a WSN is deemed unconnected, if the sink node does not belong to the giant connected component. Considering that practical sink node placement is often constrained by environmental factors [12], it is crucial to treat the sink node separately and consider its placement in the connectivity analysis of a random WSN under various scenarios.

**Figure 1:**
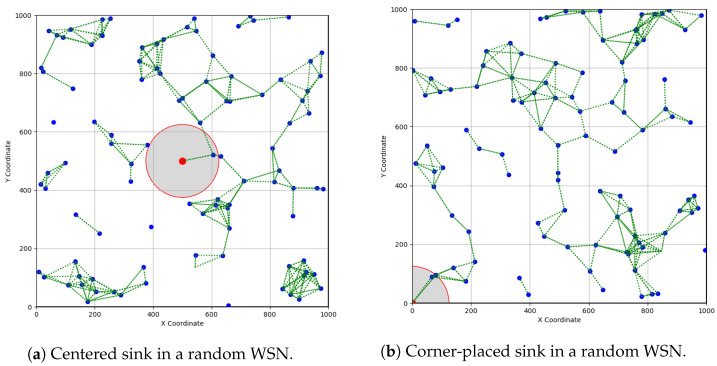
A partial connectivity comparison between a centered and a corner-placed sink in a random WSN, with sensors represented by blue dots, the sink by a red dot, and wireless links by green dashed lines. The communication range of the sink node is highlighted by a red circle with a gray shade.

This motivates us to reevaluate connectivity from a different angle, integrating the sink node and its placement for partial and full connectivity within a unified analytical framework. In practical scenarios, environmental and terrain constraints impact the placement of both the sink node and sensors [12]. Consequently, their final locations may have deviated from the ideal positions, potentially requiring corner sink placement instead of the central location. Figure 1a,b compares and contrasts the partial connectivity status of a random WSN with the same settings but differing sink placements: centered versus corner. Intuitively, center-based sink placement performs better; however, not all application scenarios allow for such convenience.

We also delve into the sensor-to-sink hop distance and its impact on network connectivity in delay-sensitive Wireless Sensor Network (WSN) applications. To this end, we introduce a maximum allowable hop distance $h_{max}$ to simulate diverse delay-sensitive application requirements. Specifically, a sensor is deemed unconnected if its hop distance to the sink exceeds the application-specific threshold $h_{max}$, thus enabling the development of an integrated framework that considers WSN parameters and application requirements for sensor-to-sink connectivity analysis under various circumstances.

### 1.1. Major Contributions

The major contributions of this paper are multiple-fold:

- Proposing an integrated analytical model for sensor-to-sink connectivity, partial connectivity, and full connectivity analysis.
- Mathematically deriving the hop distance, sensor-to-sink connectivity, full connectivity, and partial θ connectivity with a numerical analysis.
- Conducting Monte Carlo simulations to investigate the impact of various network parameters on full and partial connectivity under various circumstances.
- Examining the impact of sink node placement on the sensor connection rate and partial and full connectivity under various scenarios.
- Comparing the impact of critical network parameters on full connectivity and partial connectivity and quantifying the benefits for efficiency while fulfilling application requirements.
- Analyzing the trade-offs between energy efficiency and network connectivity and illustrating the significant advantages of energy conservation through partial connectivity in both free space and multi-fading environments.

### 1.2. Paper Organization

The remainder of this paper is organized as follows. Section 2 discusses some related works. Section 3 formulates the problem and defines the evaluation metrics. Section 4 presents the theoretical analysis. Section 5 discusses the simulation results. Finally, this work is concluded in Section 6.

## 2. Related Works

Based on an ad hoc network model, Dousee et al. [13] illustrated the considerable cost associated with achieving full connectivity and the detrimental impact of poor network properties like the transport capacity. They also showcased that a slight relaxation of the full connectivity requirement to a partial η-connectivity, where only a given fraction η<1 the nodes is connected, can significantly conserve network resources while enhancing the overall performance. Dousse et al. [14,15] extended this perspective by demonstrating that full connectivity does not scale well with the network size and that maintaining partial network connectivity leads to an optimal network throughput. Cai et al. [16] demonstrated that a large number of extra sensors are required to connect a small fraction of isolated sensor(s) for full connectivity. More specifically, they showcased that there exists a critical sensor density $\lambda_{0}$, around which the probability that at least a fraction α(α<1) of sensors are connected in the network increases sharply within a short interval of node density λ. Recently, Fu et al. [7] considered the role of the sink node in the network load distribution and presented a sink-oriented cascading model along with the MA-TOSCA algorithm to enhance WSNs’ resistance to cascading failures through topology optimization. By considering border effects based on a binary disk connectivity model, Hoyingcharoen et al. [17] developed an analytical formula to determine the expected degree of sink connectivity for homogeneous sensors that are unable to transmit directly to the sink.

Other studies have been dedicated to analyzing the hop distance (i.e., hop count) due to its close relationship with network connectivity, communication delays, and network algorithms and protocols [18,19,20,21,22,23]. Li et al. [18] derived an analytical hop count distribution (HCD) expression for a finite ad hoc network with all nodes randomly and uniformly distributed. They also introduced an equivalent area replacement method to derive the hop count distribution given an arbitrary source node and destination node pair and validated their results via simulation. Tu [19] proposed a hop count matrix recovery scheme using a decision tree methodology to recover missing items caused by attacks, and investigated the topology inference and its applications in range-free localization of sensor nodes. They transformed the hop count matrix into a classification problem, where multi-dimensional features are used for joint prediction to achieve a more accurate recovery performance, validated by their simulation results on the MATLAB R2016b platform. Huang et al. [20] proposed a self-supervised hop-count-based model (HCM) to learn and estimate hop counts based on both local and global contextual information to detect anomalies in attributed networks. The experimental results showcased the effectiveness of their approach. Liu et al. [21] proposed an algorithm employing a differential evolution algorithm to correct the estimated distance for the DV-Hop localization algorithm for error reduction. Kanwar et al. [22] proposed a framework for DV-Hop localization for displaced sensor nodes using particle swarm optimization and compared its performance with the traditional framework. They showed that their proposed method results in approximately 81% less elapsed time and 67% less energy consumption, with comparable errors. Before that, Bettstetter et al. [24] formulated the hop distance in a uniformly distributed ad hoc network, derived the one-hop and two-hop connectivity probability in closed-form expressions, and investigated the hop distance distribution between any two nodes via simulations. Dulman et al. [25] established the relationship between the hop distance and the Euclidean distance in both 1D and 2D networks. They provided an exact recursive formula for 1D networks, introduced two approximation methods for 2D networks, and showcased the effectiveness of integrating these statistical findings into existing localization algorithms to enhance performance. To address the question of ‘how many hops does it take for a packet to be relayed for a given distance’, Zhao et al. [26] conducted both probabilistic and statistical studies. They proposed an attenuated Gaussian approximation for the calculation of the conditional pdf, denoted as $f(\gamma |H_{i})$, where $H_{i}$ represents the minimum number of hops, *i*, from the source to the specific node at Euclidean distance γ. Ta et al. [27] formulated a recursive analytical equation to compute the *k*-hop connection probability for two random sensors separated by an Euclidean distance *x* to be connected in *k* hops. They validated this analysis through simulations. Recently, Li et al. [18] introduced the concept of unconditional hop count distribution (HCD) for practical networks, which eliminates the need for specifying the source-to-destination distance. They also introduced a mathematical framework, named the EARM, and derived an analytical expression for the distribution of hop counts in finite multi-hop ad hoc networks using minimum hop route mechanisms and validated the analytical results via simulations.

Furthermore, to address the inter-related challenges of coverage, connectivity, and energy efficiency, Banoth et al. [28] proposed an energy-aware distributed algorithm for maximizing coverage and achieving energy-aware connectivity by grouping sensor nodes into cover sets. The proposed algorithm maximizes the number of cover sets that take turns being active to track discrete targets and extends the lifetime of each cover set. Focusing on the *t*-sweep coverage issue, Srinivas et al. [29] proposed a 1.5-approximation approach to resolve the sweep coverage issue for a chosen point of interest, which indirectly contributes to improving the network connectivity. Haq et al. [30] designed an adaptive topology management scheme that leverages phase array antennas to create directional transmission beams in WSNs. This approach extends the network’s lifetime by conserving energy through focused transmission, enabling efficient re-establishment of communication paths via alternate links when intermediate links fail and reducing overhead and energy consumption during idle listening by other nodes. Considering a 3D heterogeneous WSN, Guo et al. [31] presented an energy-efficient coverage method that combines 3D Voronoi partitioning and the K-means algorithm to optimize node deployment and determine optimal perceptual radii for an enhanced network coverage quality. Additionally, the proposed method introduces a multi-hop communication and polling mechanism to minimize node energy consumption for improved network coverage and an extended network lifetime.

With the notable successes of machine learning in computer vision, linguistics, and control, recent research has leveraged the power of machine learning to enhance connectivity and topology maintenance in WSNs. Banerjee et al. [32] proposed an ‘RL-Sleep’ algorithm that employs reinforcement learning (RL) techniques to enhance sustainable connectivity by adaptively scheduling sleep patterns of network nodes based on their perceptions of the environment and autonomous actions (transmit, listen, or sleep). Sharma et al. [33] introduced a distributed RL algorithm based on Nash Q-Learning for sensor node scheduling to maintain coverage and network connectivity. In the proposed method, each node autonomously customizes the sensing range by learning a control policy to maximize the coverage rate while extending the network lifetime to maintain connectivity in resource-constrained WSNs. Kumar et al. [34] explored the application of RL algorithms to optimize the process of connectivity restoration in cases of network disruptions or partitions. Chandrasekar et al. [35] introduced a hybrid deep learning approach to preserve network connectivity while improving WSN coverage. The hybrid approach effectively navigates the trade-off by leveraging deep neural networks (DNNs) to monitor network conditions and RL to make real-time decisions that optimize both the coverage and lifetime. Nguyen et al. [36] presented a self-learning clustering protocol to autonomously identify neighboring nodes and the network’s topology, aiming to maintain robust and continuous network connectivity. Mirzaei et al. [37] proposed a deep-learning-based approach for establishing connectivity in mobile sensor networks, where high-traffic zones may lead to energy depletion and network partitioning. In their approach, beamforming strategies were employed to enhance the connectivity of isolated sensors and reduce the energy by up to 30% in partition healing while ensuring the network throughput via simulation.

## 3. Modeling, Problem Formulation, and Definitions

### 3.1. Modeling and Problem Formulation

Two common models, including the network deployment model for spatial sensor distribution and the disk communication model for wireless communication channel between sensors, are adopted [12,16,25,38].

**Figure 2:**
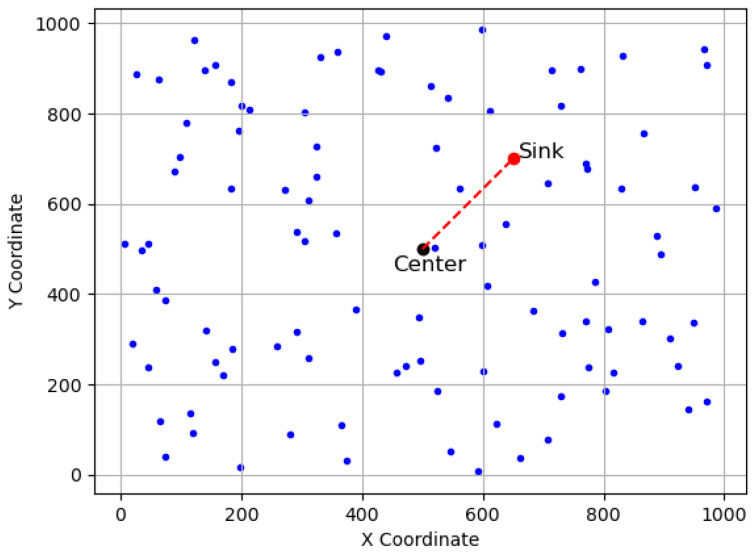
Modeling a randomly deployed Wireless Sensor Network (WSN) with a skewed sink node in a square candidate region of side length L=1000, where the candidate sink position is at the network center (L/2,L/2). Sensors are depicted as blue dots, the sink as a red circle, and the center as a black circle.

In the network model, a number of *N* sensors are randomly and independently deployed in a bounded two-dimensional square field of interest *(FoI)* with side length *L* and area A=L∗L, as shown in Figure 2. The sink node is located at a position $\left(x_{c}+\sigma_{x},y_{c}+\sigma_{y}\right)$, where $\left(x_{c},y_{c}\right)$ is the sink’s candidate position and $\sigma_{x}$ and $\sigma_{y}$ are the skewed or deviated distance in the *x* and *y* dimensions.

Note that $\sigma_{x}$ and $\sigma_{y}$ are introduced here to simulate the impact of environmental and terrain factors during deployment. For instance, a set of sensors is intended to be deployed in a designated region centered at the sink node; however, due to wind and/or terrain effects, they might eventually deviate from their intended positions by a distance of $\sigma_{x}$ and $\sigma_{y}$, respectively. Consequently, the sensors are deployed at an offset distance of $\sigma_{x}$ and $\sigma_{y}$ from the centered sink node. Here, $\sigma_{x}$ and $\sigma_{y}$ represent the relative offset distance between the sink node and sensors, and a Cartesian system can be constructed accordingly, as shown in Figure 2.

In the disk communication model, any two sensors $s_{i}\left(x_{i},y_{i}\right)$ and $s_{j}\left(x_{j},y_{j}\right)$ can communicate with each other directly if the Euclidean distance between them is no more than the communication range $r_{c}$ [39]. Namely, $s_{i}$ and $s_{j}$ can communicate directly if and only if:

(1) $$\sqrt{{\left(x_{i}-x_{j}\right)}^{2}+{\left(y_{i}-y_{j}\right)}^{2}}\leq r_{c},$$

and thus are called neighbors.

A sensor is said to be connected if and only if there exists a communication path to reach the sink node directly or in a multi-hop fashion. Any two sensors can also communicate in a multi-hop way if there exists a communication path connecting them.

### 3.2. Definitions

To investigate the connectivity problem in a random WSN, we define the following metrics for performance evaluation:

- Hop Distance P(h|d): It is defined as the probability that a random sensor at an Euclidean distance of *d* to the sink has an *h*-hop communication path to the sink.
- Sensor-to-Sink Connectivity $P_{\mathrm{con}}\left(x,y\right)$: It is defined as the probability that a sensor at position (x,y) is connected to the sink within $h_{max}$ hops, where $h_{max}$ is the maximum allowable hop distance specified in a WSN application. Namely, $P_{con}\left(x,y\right)$ is the probability that there exists a $h(h\leq h_{max})$ hop communication path between the sensor at (x,y) and the sink.
- Partial θ-Connectivity: Given a maximum allowable hop distance $h_{max}$ and θ(θ<100%) in a WSN application, the partial θ-connectivity is defined as the probability that at least a fraction of θ sensors are connected to the sink within $h_{max}$ hops.
- Sensor Connection Rate (SCR: α): It is defined as the percentage of connected sensors in a WSN. Mathematically speaking, it is expressed as $\alpha =\frac{N_{c}}{N}*100\%$, where $N_{c}$ is the number of sensors that can form a communication path within $h_{max}$ hops to the sink and *N* is the total number of sensors in the WSN.
- Normalized Energy Consumption Ratio (NECR): It is defined as the ratio of energy consumption $E_{tx}\left(l,r_{c}\right)$ to the baseline $E_{tx}\left(l,r_{0}\right)$, where $r_{0}$ represents the baseline communication range.

## 4. Theoretical Derivation and Analysis

### 4.1. Minimum Node Degree Analysis

In the considered network model, the probability that *m* sensors reside in the communication area $\pi r_{c}^{2}$ of an arbitrary sensor $s_{i}$ follows the Poisson distribution.

(2) $$P\left(m,\lambda \pi r_{c}^{2}\right)=\frac{{\left(\lambda \pi r_{c}^{2}\right)}^{m}}{m!}*e^{-\lambda \pi r_{c}^{2}},$$

where λ=N/A.

The probability that there are no neighbors within a random sensor $s_{i}$’s communication area of $\pi r_{c}^{2}$ can be derived as $P\left(0,\lambda \pi r_{c}^{2}\right)=e^{-\lambda \pi r_{c}^{2}}$.

Let $d_{i}$ represent the node degree of sensor $s_{i}$. The probability that $s_{i}$ is not isolated, i.e., there exists at least one neighboring sensor within its communication range $r_{c}$, denoted by $P(d_{i}\geq 1)$, can be calculated as:

(3) $$\begin{array}{ccc} P(d_{i}\geq 1) & = & 1-e^{-\lambda \pi r_{c}^{2}}. \end{array}$$

$P(d_{i}\geq 1)$ provides the probabilistic lower bound for sensor connectivity, and critical network parameters can be calculated in given conditions accordingly. For example, given a required probability of ξ(ξ≤1) that no node is isolated in a WSN application, the average node degree $d_{avg}$ can be derived as $\xi =1-e^{-d_{avg}}$, and we have

(4) $$\begin{array}{ccc} d_{avg} & = & -\ln(1-\xi ). \end{array}$$

**Figure 3:**
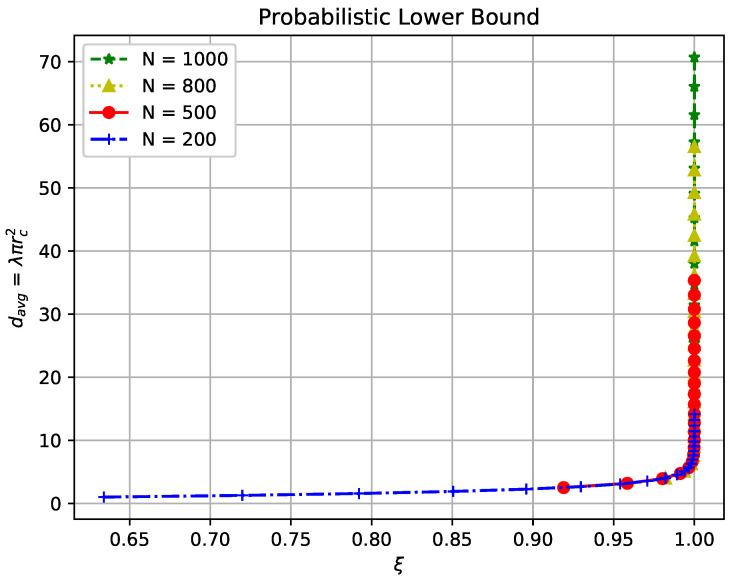
Critical average node degree $d_{avg}=\lambda \pi r_{c}^{2}$ with varying ξ for *N* = 200, 500, 800, 1000, respectively.

Figure 3 illustrates the critical average node degree $d_{avg}=\lambda \pi r_{c}^{2}$ with varying ξ for *N* = 200, 500, 800, 1000, respectively. We observe in the figure that, given ξ, the required average node degrees for all four study cases are overlapped and they increase as ξ increases. This indicates that the critical average node degree plays a determining role in fulfilling the lower bound of sensor connectivity. Moreover, when ξ approaches 1, the required average node degree increases sharply. This confirms the necessity of investigating the partial connectivity problem in WSNs.

Let $d_{min}$ represent the minimum node degree in the network and assume statistical independence. The probability that none of the *N* sensors are isolated, denoted by $P(d_{min}\geq 1)$, is derived as [40]:

(5) $$P\left(d_{min}\geq 1\right)={\left(1-e^{-\lambda \pi r_{c}^{2}}\right)}^{N}.$$

**Figure 4:**
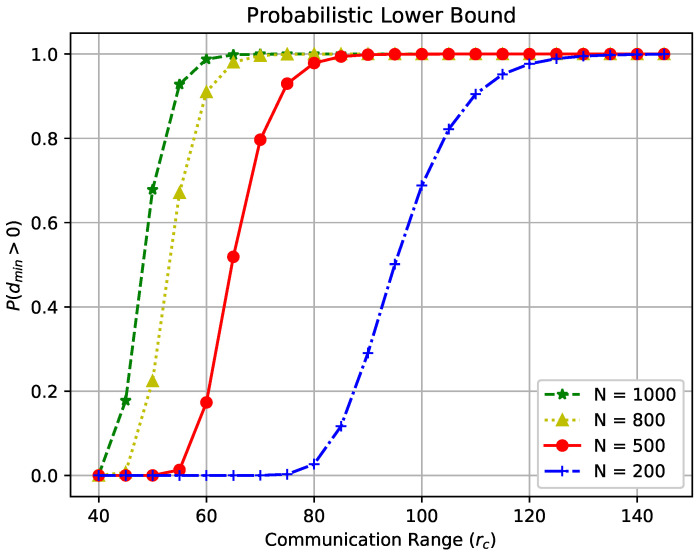
$P(d_{min}\geq 1)$ with varying $r_{c}$ from 40 m to 150 m for *N* = 200, 500, 800, 1000, respectively.

Figure 4 illustrates the probability of having no isolated sensor with the considered WSN settings, while varying the communication range $r_{c}$ from 40 m to 150 m for *N* = 200, 500, 800, 1000, respectively. It can be observed that there exists a critical communication range around which the probability $P(d_{min}\geq 1)$ rapidly transitions from 0 to 1 within a short interval of communication ranges, and the length of these intervals varies with the node density. For instance, in WSNs with a high node density (i.e., N=1000), the interval is much shorter than that in low-density networks (e.g., N=200). Detailed results for this analysis are presented in Table 1.

### 4.2. Sensor-to-Sink Connectivity

In this section, we derive and analyze the delay-oriented sensor-to-sink connectivity, the full connectivity, and the partial θ-connectivity according to the network models and definitions in Section 3.

**Theorem** **1.** *Suppose $h_{max}$ ($h_{max}>0$) is the maximal allowable hop distance for timely communication in a given WSN application. Let $P_{con}\left(x,y\right)$ be the probability that a sensor at position (x,y) is connected to the sink at position $\left(x_{0},y_{0}\right)$ within $h_{max}$ hops in the considered network model with node density λ and disk communication range $r_{c}$. $P_{con}\left(x,y\right)$ can be derived as:*

(6) $$\begin{array}{c} P_{con}\left(x,y\right)=\Sigma_{h=1}^{h_{max}}P\left(h|\sqrt{{\left(x-x_{0}\right)}^{2}+{\left(y-y_{0}\right)}^{2}}\right), \end{array}$$

*where $P\left(h|\sqrt{{\left(x-x_{0}\right)}^{2}+{\left(y-y_{0}\right)}^{2}}\right)=\left(1-e^{-2\lambda \int_{d-r_{c}}^{d+r_{c}}P\left(h-1|l\right)l\theta dl}\right)*(1-\sum_{k=1}^{h-1}$$P(k|\sqrt{{\left(x-x_{0}\right)}^{2}+{\left(y-y_{0}\right)}^{2}}))$.*

**Proof.**  The Euclidean distance from the sensor at position (x,y) to the sink is computed as $d=\sqrt{{\left(x-x_{0}\right)}^{2}+{\left(y-y_{0}\right)}^{2}}$. P(h|d) is the probability that sensor *s* at distance *d* is *h* hops away from the sink. Then, the probability that the sensor s(x,y) at distance *d* to the sink is connected within the maximum allowable hop distance $h_{max}$ is computed as:

(7) $$P_{con}\left(x,y\right)=\Sigma_{h=1}^{h_{max}}P\left(h|d\right).$$

Without loss of generality, we build a Cartesian coordinate system and set the sink at the origin O(0,0), shifting the Cartesian coordinate system if $x_{0}\neq 0$ or $y_{0}\neq 0$. According to the disk communication model, the probability that the sensor *s* is connected to the sink in one hop is given by:

(8) $$P\left(h=1|d\right)=\left\{\begin{array}{cc} 1, & d\leq r_{c};\\ 0, & d>r_{c}. \end{array}\right..$$

**Figure 5:**
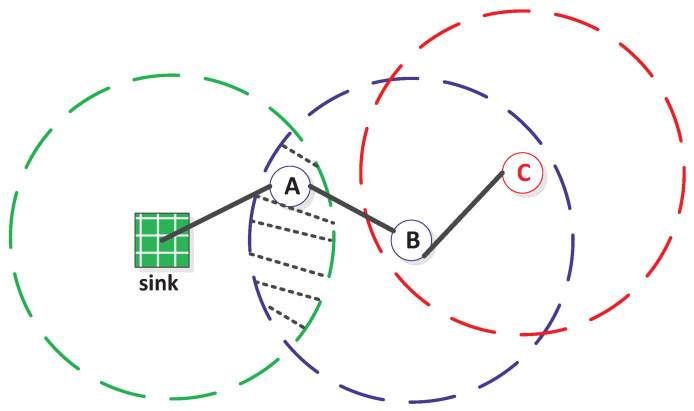
Illustration of a multi-hop communication path sink&→A→B→C, where A, B, and C represent three sensors. The overlapped communication range of the sink and the 2nd-hop sensor B is represented by the slashed area where sensor A is located and acts as a relay between the sink and sensor B. The Euclidean distance between the sink and sensor B is denoted as *d*, where $r_{c}<d\leq 2r_{c}$.

When $d>r_{c}$, the sensor can only form a multi-hop communication path to the sink. On the one hand, it can reach the sink in two hops like sensor *B* (using *A* as a relaying node), as illustrated in Figure 5. Specifically, the required conditions for a sensor like *B* to be two-hop-connected are:

- The Euclidian distance from the sensor to the sink must be between $r_{c}$ and $2*r_{c}$, i.e., $r_{c}<d<2r_{c}$.
- There should exist at least one sensor like *A* in the intersectional area between the communication disk centered at the sink and that centered at the sensor, i.e., the shaded area as shown in Figure 5.

Hence, the two-hop connection probability P(h=2|d) for a sensor at distance *d* to the sink can be derived as below:

(9) $$P\left(h=2|d\right)=\left\{\begin{array}{cc} 1-e^{-\lambda S}, & r_{c}<d\leq 2r_{c};\\ 0, & \;\mathrm{otherwise}, \end{array}\right.$$

where *S* is the area of the shaded intersectional communication area between the sink and the considered sensor. *S* was derived in [27] as:

(10) $$S=2r_{c}^{2}\arcsin\left(\sqrt{\left(1-\frac{d^{2}}{4r_{c}^{2}}\right)}\right)-dr_{c}\sqrt{\left(1-\frac{d^{2}}{4r_{c}^{2}}\right)}.$$

In Equation (9), $e^{-\lambda S}$ is the probability that no sensor is deployed in the shaded area *S* to serve as the relay sensor between sensor *s* and the sink. Thus, $1-e^{-\lambda S}$ is the probability that at least one sensor exists in the shaded area *S* to connect sensor *s* with the sink in two hops.

On the other hand, when $r_{c}<d<2r_{c}$, it is possible that the sensor *s* is connected to the sink in h≥3 hops when there is no sensor located in the shaded intercommunication area. In order to derive the probability that sensor *s* at distance *d* is connected to the sink at h(h≥3) hops, i.e., P(h|d) for h≥3, it is necessary to understand two necessary conditions:

- The sensor should *not* be connected to the sink within (h−1) or less hops;
- There should exist at least one relaying sensor in its communication range and the relaying sensor should be connected to the sink in *exactly* (h−1) hops.

Considering the first condition, the probability that a sensor *s* at distance *d* is connected to the sink within (h−1) or less hops is derived as $\sum_{k=1}^{h-1}P\left(k|d\right)$. Thus, the probability that it is *not* connected within (h−1) is computed as $1-\sum_{k=1}^{h-1}P\left(k|d\right)$.

As to the second condition, the probability that at least one sensor is located in sensor *s*’s communication range and the sensor is connected to the sink in exactly (h−1) hops can be computed as $\left(1-e^{-2\lambda \int_{d-r_{c}}^{d+r_{c}}P\left(h-1|x\right)x\theta dx}\right)$, where $\theta =\arccos\frac{\left(d^{2}+l^{2}-r_{c}^{2}\right)}{2ld}$ and $d-r_{c}<l<d+r_{c}$. Consequently, the probability P(h|d) that a sensor at distance *d* is connected at a hop distance of h(h≥3) can be computed recursively via [27,41]:

$$P\left(h|d\right)=\left(1-e^{-2\lambda \int_{d-r_{c}}^{d+r_{c}}P\left(h-1|l\right)l\theta dl}\right)*\left(1-\sum_{k=1}^{h-1}P\left(k|d\right)\right).$$

Finally, given $h_{max}$, the probability that a sensor at position (x,y) with distance $d=\sqrt{{\left(x-x_{0}\right)}^{2}+{\left(y-y_{0}\right)}^{2}}$ is connected to the sink is derived as:

(11) $$\begin{array}{ccc} P_{con}\left(x,y\right) & = & \sum_{h=1}^{h_{max}}P\left(h|\sqrt{{\left(x-x_{0}\right)}^{2}+{\left(y-y_{0}\right)}^{2}}\right). \end{array}$$

□

**Figure 6:**
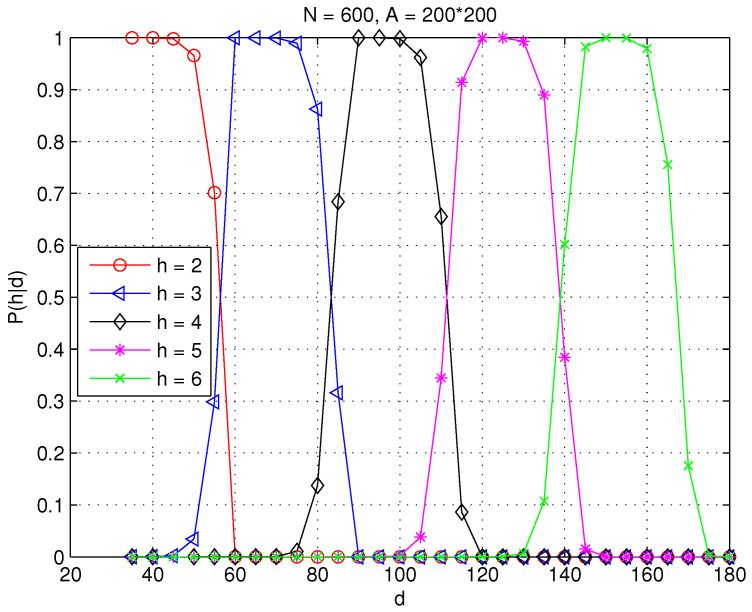
The probability that a sensor at distance *d* is connected to the centered sink at *h* hops in a WSN with N=600, $r_{c}=30$, and $A=200^{2}$ square meters.

**Figure 7:**
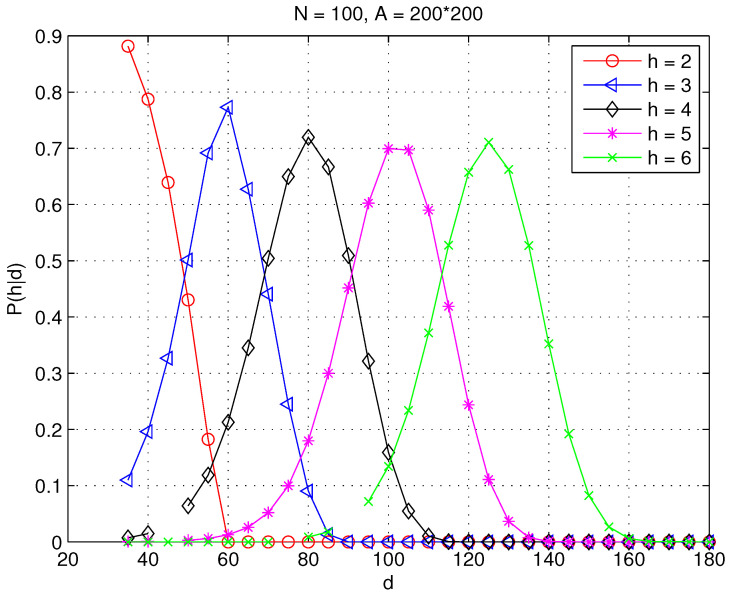
The probability that a sensor at distance *d* is connected to the centered sink at *h* hops in a WSN with N=100, $r_{c}=30$, and $A=200^{2}$ square meters.

Figure 6 and Figure 7 plot the numerical results on P(h|d) for h=2,⋯,6 in random WSNs. Specifically, $r_{c}$ is fixed at 30 and the area of interest is fixed at A(−100≤x≤100,−100≤y≤100) with area $\left|A\right|=200^{2}$ square meters in both network settings. N=600 and N=100 are selected to generate different network topologies with average node degrees of 42 and 7 in Figure 6 and Figure 7, respectively. It can be seen clearly that the probability that a sensor with a certain distance to the sink is connected by *h* hops differs under different network settings.

**Figure 8:**
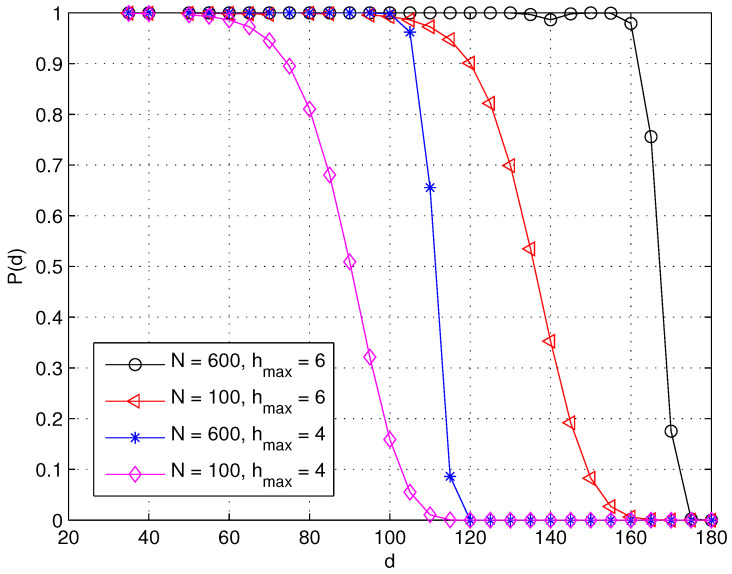
The probability that a sensor at distance *d* is connected to the centered sink within $h_{max}$ hops.

Figure 8 depicts the probability P(d) for $h_{max}=4$ and $h_{max}=6$ for N=100 and N=600, respectively. We observe that sensors at the same Euclidean distance from the sink are less likely to be connected in a weakly connected WSN. For instance, when other network settings remain the same, $P_{N=100}\left(d\right)<P_{N=600}\left(d\right)$. Furthermore, sensors located farther away from the sink have reduced sensor connectivity, i.e., $P\left(d_{1}\right)<P\left(d_{2}\right)$ if $d_{1}>d_{2}$ in a *partially* connected WSN. This is different from the sensor connectivity in a fully connected WSN, where P(d)=1 regardless of the sensor’s Euclidean distance *d* to the sink.

### 4.3. Full and Partial θ Connectivity

**Theorem** **2.** *Suppose $h_{max}$ ($h_{max}>0$) is the maximal allowable hop distance for timely communication in a given WSN application. Let $P_{con}\left(h_{max}\right)$ be the full connectivity probability that all sensors are connected to the sink within $h_{max}$ hops in the considered network model with N randomly and independently deployed sensors. Assume $\left(x_{i},y_{i}\right)$ (i=1,⋯,N) is the $i_{th}$ sensor’s coordinate and the sink is located at position $\left(x_{0},y_{0}\right)$. $P_{con}\left(h_{max}\right)$ can be derived as:*

(12) $$\begin{array}{ccc} P_{con}\left(h_{max}\right) & = & \prod_{i=1}^{N}\Sigma_{h=1}^{h_{max}}P\left(h|\sqrt{{\left(x_{i}-x_{0}\right)}^{2}+{\left(y_{i}-y_{0}\right)}^{2}}\right). \end{array}$$

**Proof.**  A WSN is considered fully connected if all the *N* sensors are connected to the sink within $h_{max}$ hops according to the definition in Section 3.2. As proven in Theorem I, the probability that a sensor at position $\left(x_{i},y_{i}\right)$ is connected to a centered sink within $h_{max}$ is given by $P_{con}\left(x_{i},y_{i}\right)=\sum_{h=1}^{h_{max}}P\left(h|\sqrt{{\left(x_{i}-x_{0}\right)}^{2}+{\left(y_{i}-y_{0}\right)}^{2}}\right)=\sum_{h=1}^{h_{max}}P\left(h|d_{i}\right)=\left(1-e^{-2\lambda \int_{d-r_{c}}^{d+r_{c}}P\left(h-1|l\right)l\theta dl}\right)*\left(1-\sum_{k=1}^{h-1}P\left(k|d_{i}\right)\right)$, where $d_{i}=\sqrt{{\left(x_{i}-x_{0}\right)}^{2}+{\left(y_{i}-y_{0}\right)}^{2}}$.

Assuming statistical independence for all the sensors, the full connectivity is calculated as:

(13) $$P_{con}\left(h_{max}\right)=\prod_{i=1}^{N}P_{con}\left(x_{i},y_{i}\right).$$

Consequently,

$$\begin{array}{ccc} P_{con}\left(h_{max}\right) & = & \prod_{i=1}^{N}\Sigma_{h=1}^{h_{max}}P\left(h|\sqrt{{\left(x_{i}-x_{0}\right)}^{2}+{\left(y_{i}-y_{0}\right)}^{2}}\right)\\ & = & \prod_{i=1}^{N}\Sigma_{h=1}^{h_{max}}\left(1-e^{-2\lambda \int_{di-r_{c}}^{d_{i}+r_{c}}P\left(h-1|l\right)l\theta dl}\right)\\ & * & (1-\sum_{k=1}^{h-1}P\left(k|d_{i}\right)). \end{array}$$

□

**Theorem** **3.** *Suppose $h_{max}$ ($h_{max}>0$) is the maximal allowable hop distance for timely communication in a given WSN application. Let $P_{\theta }\left(h_{max}\right)$ be the θ connectivity probability that a fraction of θ sensors are connected to the sink within $h_{max}$ hops in the considered network model with N randomly and independently deployed sensors. Assume $\left(x_{i},y_{i}\right)$ (i=1,⋯,N) is the $i_{th}$ sensor’s coordinate and the sink is located at position $\left(x_{0},y_{0}\right)$. $P_{\theta }\left(h_{max}\right)$ can be derived as:*

(14) $$\begin{array}{ccc} P_{\theta }\left(h_{max}\right) & = & P[\frac{\Sigma_{i=1}^{N}\Sigma_{h=1}^{h_{max}}P\left(h|d_{i}\right)}{N}>\theta ], \end{array}$$

*where $d_{i}=\sqrt{{\left(x_{i}-x_{0}\right)}^{2}+{\left(y_{i}-y_{0}\right)}^{2}}$ and $P\left(h|d_{i}\right)=\left(1-e^{-2\lambda \int_{d-r_{c}}^{d+r_{c}}P\left(h-1|l\right)l\theta dl}\right)*(1-$$\sum_{k=1}^{h-1}P\left(k|d_{i}\right))$.*

**Proof.**  As proven in Theorem I, the probability that a sensor at position $\left(x_{i},y_{i}\right)$ is connected to the sink at position $\left(x_{0},y_{0}\right)$ within $h_{max}$ hops is given by $P_{con}\left(x_{i},y_{i}\right)=\sum_{h=1}^{h_{max}}$$P\left(h|\sqrt{{\left(x_{i}-x_{0}\right)}^{2}+{\left(y_{i}-y_{0}\right)}^{2}}\right)=\sum_{h=1}^{h_{max}}P\left(h|d_{i}\right)=\left(1-e^{-2\lambda \int_{d-r_{c}}^{d+r_{c}}P\left(h-1|l\right)l\theta dl}\right)*$$\left(1-\sum_{k=1}^{h-1}P\left(k|d_{i}\right)\right)$, where $d_{i}=\sqrt{{\left(x_{i}-x_{0}\right)}^{2}+{\left(y_{i}-y_{0}\right)}^{2}}$.

Assuming statistical independence for all the sensors, $\Sigma_{i=1}^{N}P_{con}\left(x_{i},y_{i}\right)$ is therefore the sum of the *N* sensors’ connectivity and $\frac{\Sigma_{i=1}^{N}P_{con}\left(x_{i},y_{i}\right)}{N}$ is the expected fraction of connected sensors. According to the definition of partial θ-connectivity defined in Section 3.2, a WSN is said to be θ connected if at least a fraction of θ sensors are connected to the sink within $h_{max}$ hops, i.e., $\frac{\Sigma_{i=1}^{N}P_{con}\left(x_{i},y_{i}\right)}{N}>\theta$. Thus, $P_{\theta }\left(h_{max}\right)$ is derived as:

(15) $$\begin{array}{ccc} P_{\theta }\left(h_{max}\right) & = & P[\frac{\Sigma_{i=1}^{N}P_{con}\left(x_{i},y_{i}\right)}{N}>\theta ],\\ & = & P[\frac{\Sigma_{i=1}^{N}\Sigma_{h=1}^{h_{max}}P\left(h|\sqrt{x_{i}^{2}+y_{i}^{2}}\right)}{N}>\theta ]. \end{array}$$

where $P\left(h|d_{i}\right)=\left(1-e^{-2\lambda \int_{d_{i}-r_{c}}^{d_{i}+r_{c}}P\left(h-1|l\right)l\theta dl}\right)*\left(1-\sum_{k=1}^{h-1}P\left(k|d_{i}\right)\right)$. □

**Figure 9:**
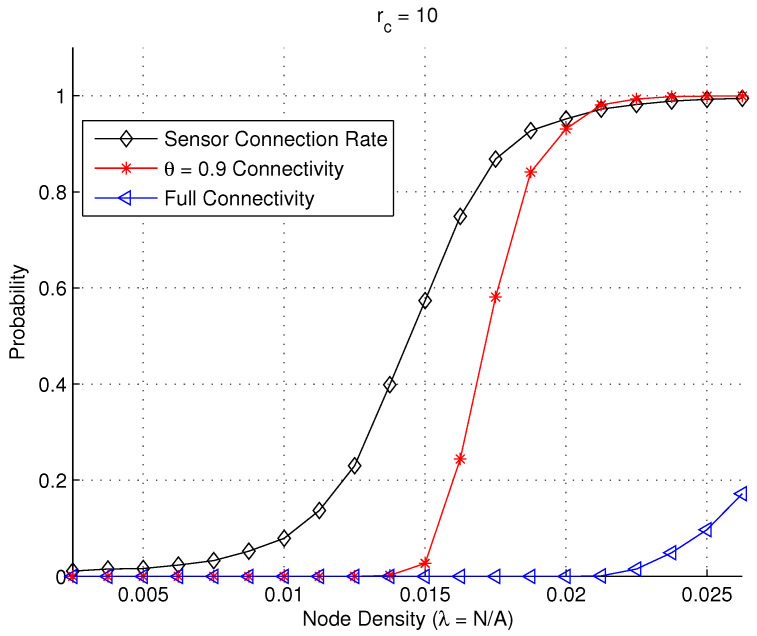
Full connectivity, partial 0.9 connectivity, and sensor connection rate in a random WSN.

Figure 9 shows the network full connectivity, partial θ=0.9 connectivity, and the average sensor connection rate for varying values of node density when the communication range is set as $r_{c}=10$ m. It is observed that the network full connectivity contrasts sharply with the partial θ=0.9 connectivity and a larger node density will be required to connect the most isolated sensors for full connectivity. This is not economic for many WSN applications that do not require full connectivity such as intrusion detection [38,42]. The results confirm the necessity of investigating the partial connectivity and examining its impact on the hop distance under various settings.

## 5. Simulation and Discussion

In this section, we conduct extensive simulations to investigate network connectivity for various WSNs. The simulation is developed in Java, and the results are aggregated and plotted in Python 3. Unless otherwise specified, the network *FoI* is set as a two-dimensional square with side length L=1000 m. All simulation results are the average of 1000 runs.

**Figure 10:**
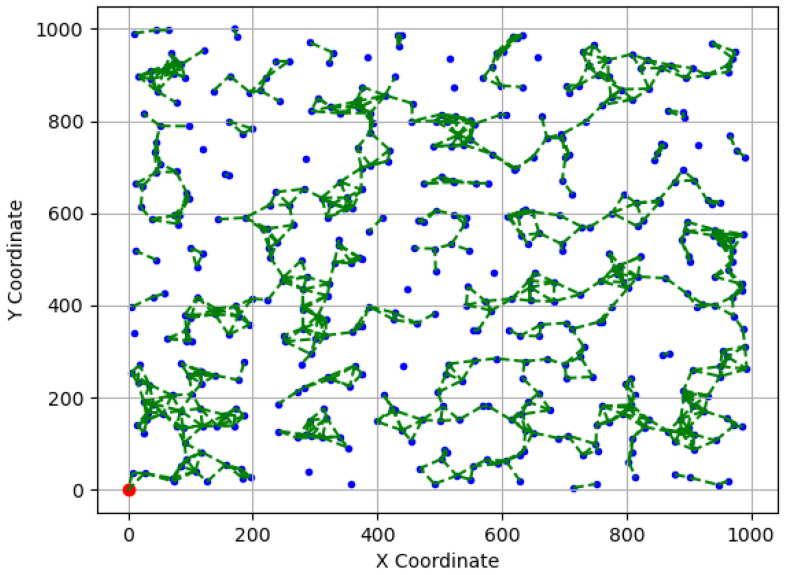
An example of a simulated WSN case (a), where sensors are depicted as blue dots and communication links as dashed green lines.

**Figure 11:**
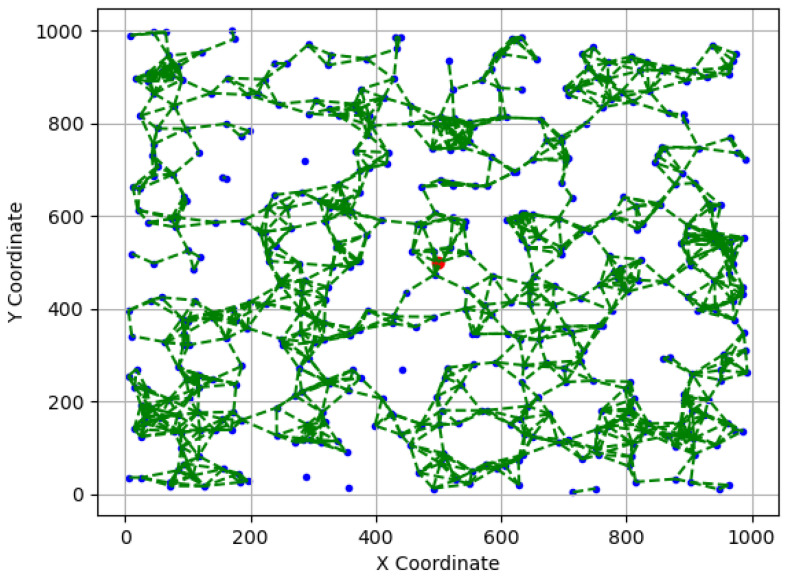
An example of simulated WSN case (b), where sensors are depicted as blue dots and communication links as dashed green lines.

Figure 10 and Figure 11 showcase two examples of simulated WSN cases: (a) A simulated WSN case with a bordered sink, where N=500, rc=50, and L=1000. (b) A simulated WSN case with a centered sink, where N=500, rc=65, and L=1000. In both figures, sensor nodes are denoted as blue dots, while the sink node is depicted as a red circle. The communication links are represented by green dashed lines.

### 5.1. Impact of Communication Range $r_{c}$

**Figure 12:**
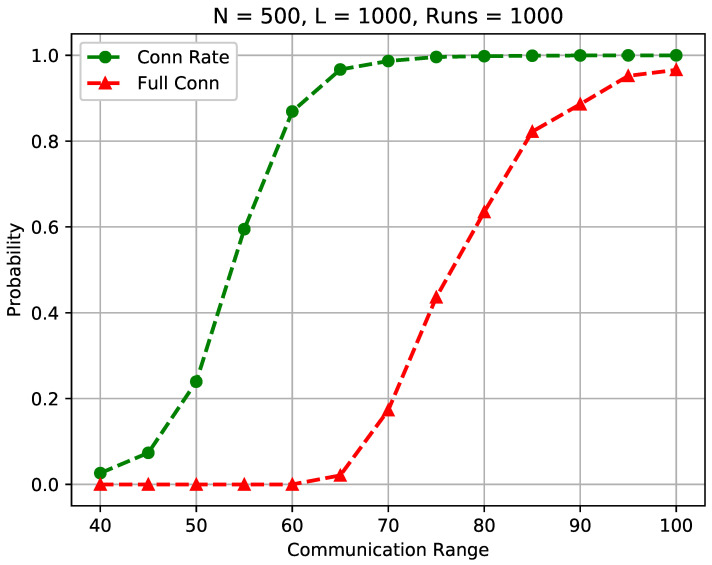
Connection rate vs. full connectivity in a random WSN.

Figure 12 compares the sensor connection rate and the full connectivity when the communication range is varied from 40 to 100 m while other network settings remain the same.

We observe in the figure that the sensor connection rate differs significantly from the full connectivity. For instance, when $r_{c}=65$, the full connectivity is low at 2.2%, while the sensor connection rate is high at 96.35%. In addition, to achieve a high probability of full connectivity (say 97.6%), the communication range should be set above 100 m in the considered conditions. On the other hand, 96.35% sensors are well connected to the BS when the communication range is only 65 m as indicated in the figure. Increasing the communication range from 65 m to 100 m will introduce an extremely higher energy consumption and communication interference. According to the free space model [2,43], all sensors need to spend approximately $\frac{100^{2}-65^{2}}{65^{2}}=$ 1.367 times more energy to connect less than 4% of isolated sensors, while $\frac{100^{4}-65^{4}}{65^{4}}=$ 4.60 times more energy will be required if following the multipath fading model [2,43]. Further in-depth analysis is provided in Section 5.4.

The results support our claim that the literature research results on achieving full connectivity in random WSNs cannot be applied to many real-life WSN designs and sensor deployments, as much larger communication ranges and radio powers are required to connect a small fraction of remote sensors, resulting in a significantly higher power consumption rate and network lifetime reduction.

### 5.2. Centered Sink vs. Bordered Sink

**Figure 13:**
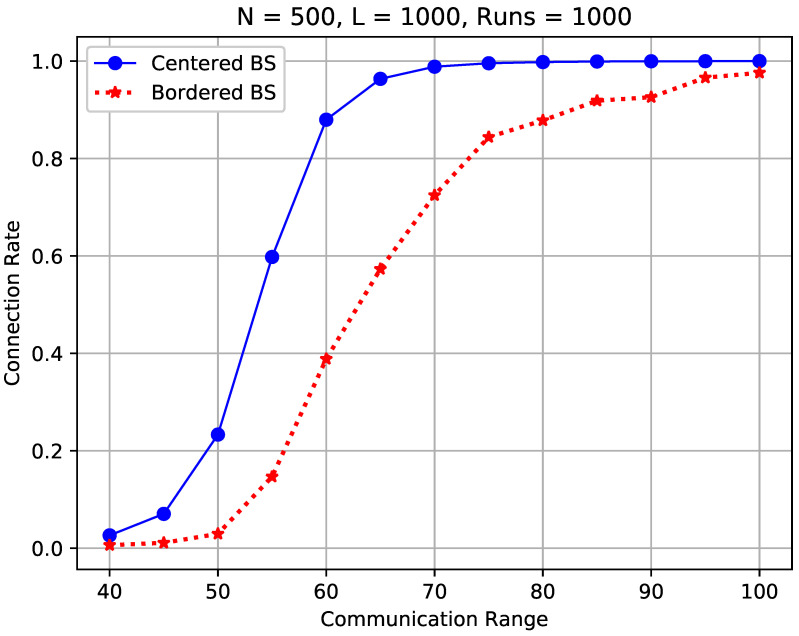
Sensor connection rate of a WSN with a centered sink vs. its counterpart with a bordered sink.

Figure 13 compares the sensor connection rate of the considered WSNs with a centered sink and a bordered sink. In this study, 500 sensors were randomly scattered in the *FoI* for all cases, and the communication range was varied from 40 m to 100 m to simulate different ranges of sensors.

In the figure, we observe a noticeable difference between the two studied WSNs in terms of the sensor connection rate. For example, to achieve a 95% sensor connection rate, the communication range should be 70 m in the WSN with a centered sink, while it should be increased to 100 m for the WSN with a bordered sink, with the other network parameters remaining the same. We also observe that the sensor connection rate of a WSN with a centered sink is always better than its counterpart with a bordered sink. For instance, the sensor connection rate drops from 98.8% to 72.5% when the sink is moved from the center to the border while $r_{c}=70$ m. This is because when the sink is placed on the border, the distance to sensors that are close to the other side increases significantly, which makes it less probable for the remote sensors to establish a communication path to reach the sink.

**Figure 14:**
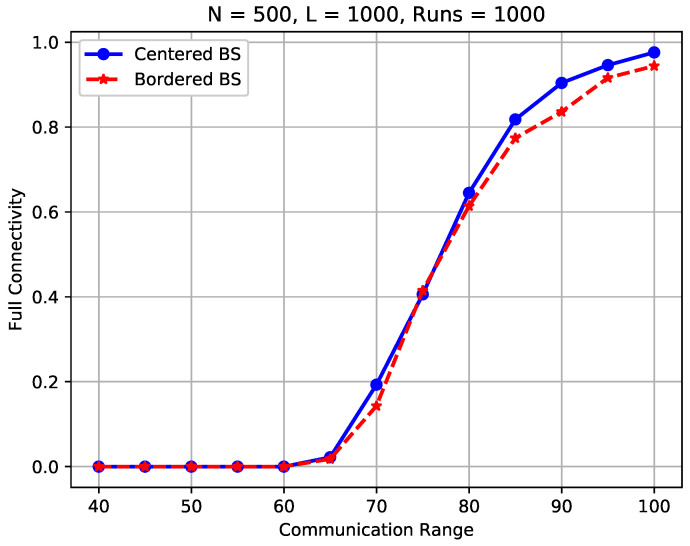
Full connectivity of a WSN with a centered sink vs. counterpart with a bordered sink.

Figure 14 compares the full connectivity of the same experiments as depicted in Figure 13. Different findings are uncovered. To be specific, the full connectivity of the studied WSNs with a centered sink nearly overlaps with the counterparts of a bordered sink, as shown in Figure 14. This means, the position of the sink has negligible influence on the full connectivity in the considered WSNs. This is because in a fully connected WSN, all sensors including the sink should be connected, no matter where the node is located. This also explains why very little work has taken into consideration the impact of sink placement on the full connectivity of random WSNs in the current literature.

The results confirm our claim that the placement of the sink has an essential impact on WSN partial connectivity in terms of the sensor connection rate.

### 5.3. Impact of Skewed Distance of Sink Placement

**Figure 15:**
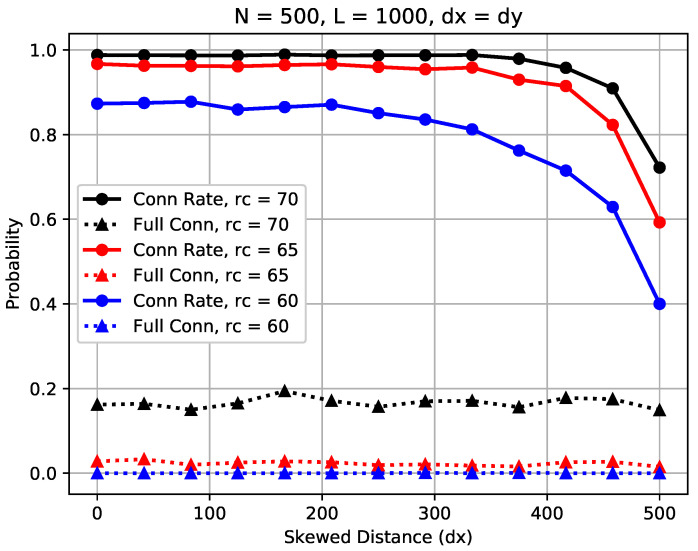
Impact of skewed distance $d_{x}$ of sink node on the sensor connection rate and full connectivity, where $d_{x}=d_{y}$.

In this section, we investigate the impact of *relative* skewed or deviated distance, denoted as $d_{x}$ and $d_{y}$ as defined in Section 3, on the network connectivity. For simplicity of analysis, we assume $d_{x}=d_{y}$. Figure 15 illustrates the impact of $d_{x}$ and $d_{y}$ on the sensor connection rate and the full connectivity in randomly scattered WSNs where N=500, L=1000 for $r_{c}=60,65$, and 70 m, respectively.

On the one hand, we observe in the figure that the skewed distance has a noticeable impact on the sensor connection rate for all three considered communication ranges. We also observe that there exists a critical skewed distance $d_{c}$, around which the sensor connection rate in the network drops quickly from the upper bound to the lower bound, and the critical skewed distance differs when network settings vary. For $r_{c}=70$ m, as shown in the figure, when the skewed distance $d_{x}$ increases from 0 to 250 m, the sensor connection rate is constant; however, it drops quickly when $d_{x}$ exceeds 250 m. On the other hand, it is shown that the skewed distance $d_{x}$ has little impact on the full connectivity. This is because all sensors, regardless of their locations, should be connected in a fully connected WSN.

The results show that randomly deployed WSNs can tolerate some extent of the skewed distance between the sink and the sensors for partial connectivity. Furthermore, with the given network settings, there exists a critical threshold skewed distance, within which the sensor connection rate is the same and beyond which it drops quickly.

### 5.4. Energy Efficiency and Trade-Off Analysis

To analyze energy efficiency and examine tradeoffs in a WSN, we adopted the widely accepted radio energy dissipation model introduced by Heinzelman et al. [2,43] for both free space ($d^{2}$) and multipath fading ($d^{4}$) environments. Assuming a transmission range of $r_{c}$, the sending sensor consumes energy as follows to transmit an *l*-bit message:

(16) $$E_{tx}\left(l,r_{c}\right)=\left\{\begin{array}{cc} l\cdot E_{elec}+l\cdot \epsilon_{fs}\cdot r_{c}^{2}, & \mathrm{free}\;\mathrm{space}\\ l\cdot E_{elec}+l\cdot \epsilon_{mp}\cdot l\cdot r_{c}^{4}, & \mathrm{multi}-\mathrm{path} \end{array}\right.$$

where $E_{elec}$ is the electronics energy and $\epsilon_{fs}$ and $\epsilon_{mp}$ are the amplifier energies. The model can be applied to various WSN applications by calibrating the parameters appropriately. In the subsequent analysis, we assume the parameters $E_{elec}=50$ nJ/bit, $\epsilon_{fs}=10$ pJ/bit/m${}^{2}$, and ϵ=0.013 pJ/bit/m${}^{4}$ as used in [2].

**Figure 16:**
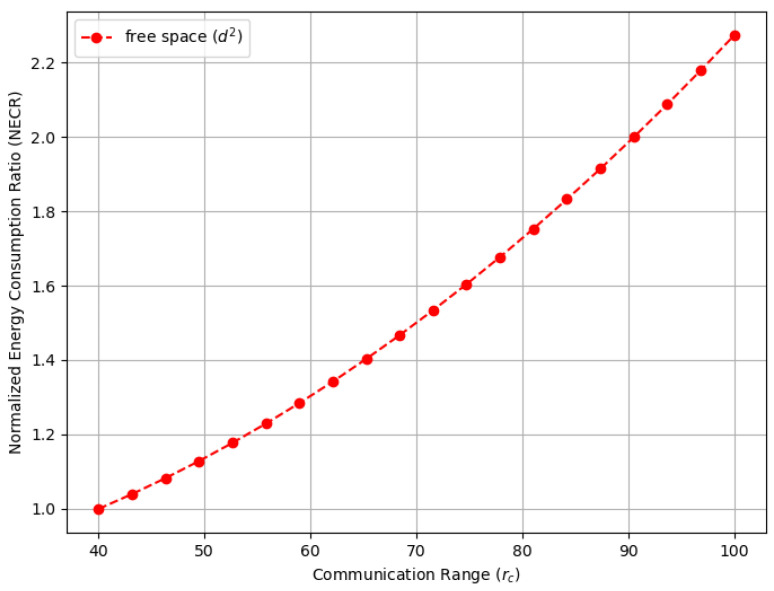
The normalized energy consumption ratio (NECR) for the free space environment in a random WSN with a varying communication range ($r_{c}$).

**Figure 17:**
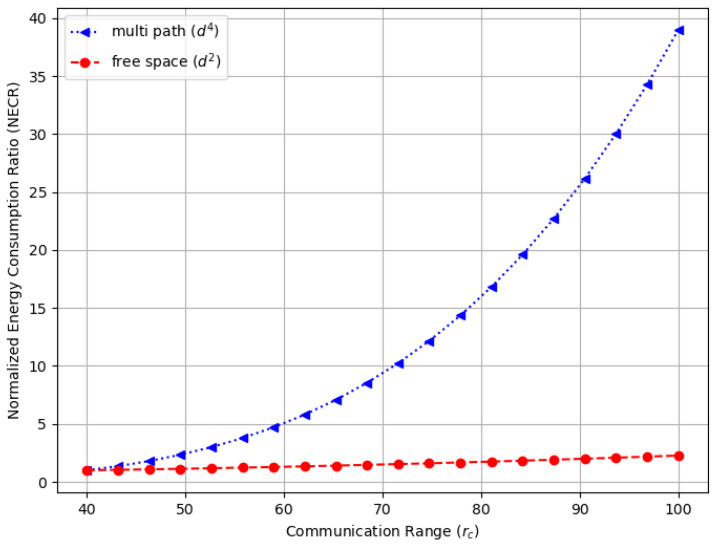
The normalized energy consumption ratio (NECR) for both free space and multi-fading environments in a random WSN with a varying communication range ($r_{c}$).

Figure 16 illustrates the normalized energy consumption ratio (NECR) for the free space environment, while Figure 17 compares the NECR in free space with multi-fading environments. In both figures, the sensors’ communication range $r_{c}$ varies from 40 to 100 m, and the NECR is defined as the ratio of energy consumption $Etx(l,r_{c})$ to the baseline $E_{tx}\left(l,r_{0}\right)$, where $r_{0}$ is set to 40 m. We observe that in both figures, the Energy Consumption Ratio increases rapidly, more than doubling in the free space environment and increasing up to 40 times in the multi-path fading scenario as the communication range extends from 40 to 100 m while other settings remain the same. Note that the data for the free space in Figure 16 are the same as those in Figure 17, which are provided for comparison purposes.

**Figure 18:**
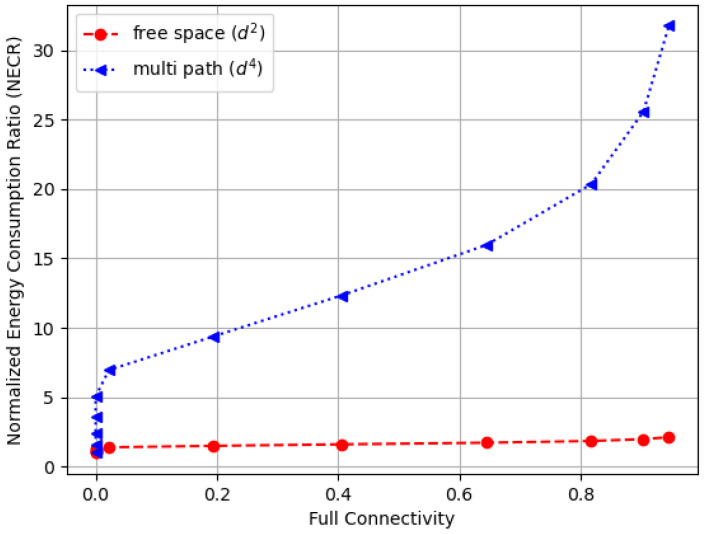
The normalized energy consumption ratio (NECR) for multi-fading environments in a random WSN with varying full connectivity requirements.

**Figure 19:**
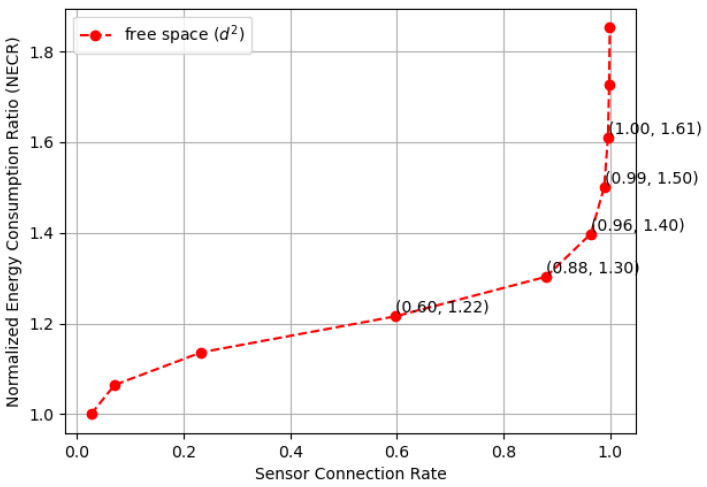
The normalized energy consumption ratio (NECR) for both free space and multi-fading environments in a random WSN with varying sensor connection rate requirements.

**Figure 20:**
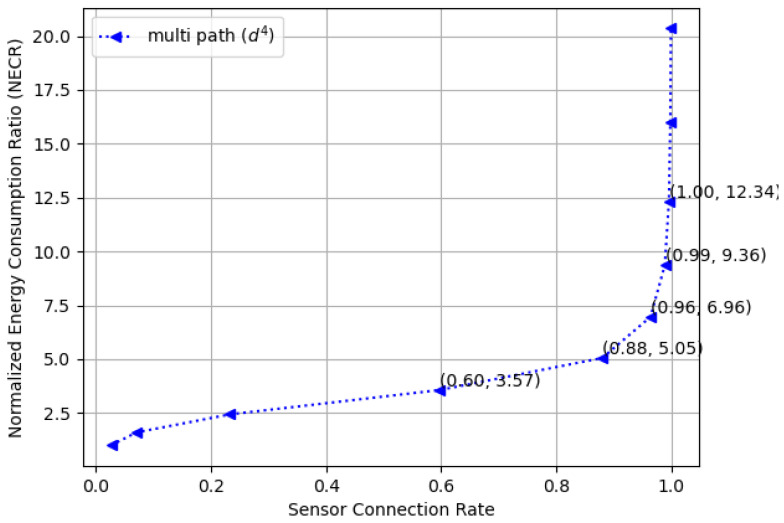
The normalized energy consumption ratio (NECR) for multi-fading environments in a random WSN with varying sensor connection rate requirements.

Figure 18 compares the normalized energy consumption ratio (NECR) in both free space and multi-fading environments while varying the full connectivity requirements, and similar trends were observed. Moreover, Figure 19 and Figure 20 illustrate the tradeoffs between the sensor connection rate and the normalized energy consumption ratio (NECR) for free space and multipath fading, respectively. We observe that to increase the sensor connection rate slightly from 96% to 100%, an additional 21% and 538% more energy are required for free space and multipath fading, respectively, while keeping other parameters constant.

This validates our claim that pursuing full connectivity introduces significant demands on network resources and dramatically decreases the energy efficiency of a WSN. Corresponding data values are also provided in Table 2 for a detailed comparison.

## 6. Conclusions

Through mathematical modeling, theoretical analysis, and simulation evaluation, we have drawn the following conclusions. First, full connectivity is not an appropriate requirement for many real-life Wireless Sensor Network (WSN) applications to adopt due to the significant cost of precious network resources and reduced energy efficiency. Second, a pre-defined sensor connection rate for partial connectivity is appropriate for most WSN performance evaluations and should be adopted to direct real-life WSN design, deployment, and implementation. Third, the placement of the sink node and its potential skewed distance to all sensors have an essential impact on partial connectivity in terms of the sensor connection rate, which should be taken into consideration. This paper provides insight into the definition of appropriate network metrics and into the selection of critical network parameters for real-life WSN design and implementation. In the future, we plan to address partial connectivity along with coverage requirements for various applications and investigate their trade-offs. Additionally, we intend to explore available empirical WSN datasets and apply data science and machine learning techniques for predictive modeling, pattern recognition, and performance evaluations and comparisons.

## Figures and Tables

**Table 1:** Critical communication range for varying $P(d_{min}\geq 1)$ when $N_{1}=200$, $N_{2}=500$, $N_{3}=800$, $N_{4}=1000$, respectively.

$r_{c}$	45	50	55	60	65	70	75	80	85	90	95	100	105	110	115	120
$N_{1}=200$	0.00	0.00	0.00	0.00	0.00	0.00	0.00	0.03	0.12	0.29	0.50	0.69	0.82	0.90	0.95	0.98
$N_{2}=500$	0.00	0.00	0.01	0.17	0.52	0.80	0.93	0.98	0.99	1.00	1.00	1.00	1.00	1.00	1.00	1.00
$N_{3}=800$	0.01	0.22	0.67	0.91	0.98	1.00	1.00	1.00	1.00	1.00	1.00	1.00	1.00	1.00	1.00	1.00
$N_{4}=1000$	0.18	0.68	0.93	0.99	1.00	1.00	1.00	1.00	1.00	1.00	1.00	1.00	1.00	1.00	1.00	1.00

**Table 2:** Impact of the communication range ($r_{c}$) on the sensor connection rate and full connectivity.

Communication Range (*r_c_*)	NECR ($d^{2}$)	NECR ($d^{4}$)	Sensor Connection Rate	Full Connectivity
40	1.00	1.00	0.026388	0
45	1.06439394	1.60063353	0.070502	0
50	1.13636364	2.43859649	0.233284	0
55	1.21590909	3.56944444	0.597838	0
60	1.3030303	5.0545809	0.879652	0
65	1.39772727	6.96125731	0.963496	0.022
70	1.50	9.3625731	0.988434	0.193
75	1.60984848	12.33747563	0.995576	0.406
80	1.72727273	15.97076023	0.997982	0.645
85	1.85227273	20.35307018	0.99912	0.818
90	1.98484848	25.58089669	0.999606	0.904
95	2.125	31.75657895	0.99976	0.946
100	2.27272727	38.98830409	0.999916	0.976

## Data Availability

Not applicable.
